# Reply to letter from J. Finsterer and S. Zarrouk-Mahjoub

**DOI:** 10.1007/s12471-013-0510-z

**Published:** 2014-01-08

**Authors:** S. A. W. G. Dello, M. Alings

**Affiliations:** Department of Cardiology, Amphia Hospital Breda, Breda, the Netherlands

We thank Dr. Finsterer and Dr. Zarrouk-Mahjoub for their comments, in which they argue for further neurological work-up of the present case [1]. We agree that neurological assessment is an integral part of the multidisciplinary approach to patients presenting with syncope. However, the authors neglect the fact that our patient was only referred after neurological investigations, which yielded no explanations for the symptoms. Documented collapse during simultaneous registration of a normal EEG and an ECG showing ventricular tachycardia (VT) prompted referral to cardiology care. Beta-blocker treatment suppressed recurrent symptomatic non-sustained VT, after which the patient remained asymptomatic.

Ventricular arrhythmias and heart failure are known clinical symptoms of non-compaction cardiomyopathy (CMP) [2]. In our opinion, in the present case the obvious relation between VT and symptoms in the absence of EEG abnormalities leaves no place for epilepsy in the differential diagnosis. Therefore, in this particular case further in-depth neurological investigation, as suggested by the authors, does not seem useful.

In general, however, in patients with non-compaction CMP in whom a clear cardiac cause for syncope such as symptomatic non-sustained VTs cannot be found, referral to the neurologist should be considered, since thromboembolic events may occur in the setting of non-compaction CMP. In the absence of atrial fibrillation or LV dysfunction, however, the occurrence of stroke is rare [3]. Although there are no robust data to support the use of oral anticoagulation, it may be recommended in patients with impaired systolic function and or atrial fibrillation [2].
